# Comprehensive review of the impact of tris(2,3-dibromopropyl) isocyanurate (TBC or TDBP-TAZTO) on living organisms and the environment

**DOI:** 10.1007/s10653-022-01206-y

**Published:** 2022-02-01

**Authors:** Monika Bar, Konrad A. Szychowski

**Affiliations:** Department of Biotechnology and Cell Biology, Medical College, University of Information Technology and Management in Rzeszow, Sucharskiego 2, 35-225 Rzeszow, Poland

**Keywords:** TDBP-TAZTO, TBC, BCF, BFRs, Toxicity, Bioindicators, Persistent pollution

## Abstract

Tris(2,3-dibromopropyl) isocyanurate (TBC or TDBP-TAZTO) belongs to the group of brominated flame retardants (BFRs). The production of this compound is increasing due to the growing demand and wide application in electrical, electronic, musical instrument, and automotive component industries. The properties of TBC, e.g., the high octanol–air partition coefficient (Koa), high octanol–water partition coefficient (Kow), and high bioconcentration factor (BCF), indicate a possibility of its spread in aquatic and terrestrial ecosystems and bioaccumulation in living organisms. The presence of TBC has been confirmed in soil, sediments, river water, and such materials as microplastic, curtains, and e-waste devices. The compound has potential to bioaccumulate in the food chain of living organisms. TBC has been demonstrated to exert a harmful effect mainly on the nervous and endocrine systems, lungs, and liver. The possible mechanism of toxicity of the compound in the nervous system is based on the generation of oxidative stress by TBC leading to apoptosis of neuronal cells, while mitochondrial damage is considered to be responsible for changes in the respiratory organ. Moreover, the potential of mussels and earthworms to be bioindicators of TBC has been proven. Therefore, the literature review is focused on TBC properties and analysis of the identification and impact of the compound on the environment, living organisms, and human cell lines. Given the many toxic effects of TBC highlighted in the literature, there is a need for more profound research on the safety of TBC and methods for identification and degradation of this compound.

## Introduction

**Fig. 1:**
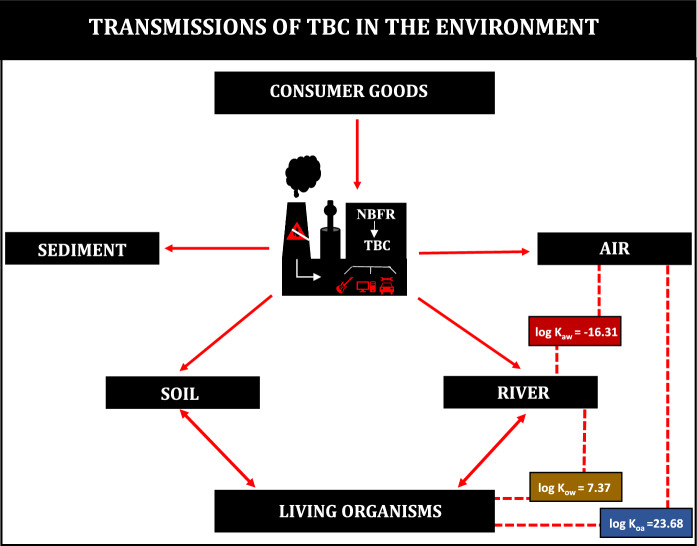
Transmissions of TBC in the environment

Flame retardants (FR) are a group of brominated and chlorinated organohalogen substances containing phosphorus, nitrogen, and inorganic compounds. Among all FR, brominated flame retardants (BFRs) are commonly used due to their low cost and high efficiency (Birnbaum & Staskal, 2004). The mechanism of action of BFRs is based on blocking the generation of flammable gases by decomposition thereof at a temperature approx. 50 °C below the combustion temperature of the host polymer (Campanale et al., 2020; Rahman et al., 2001). These results in the targeted use of BFRs in fire prevention in electrical and electronics devices and construction, textile, and transport industries (Campanale et al., 2020). BFRs are present at a level of 5–30% in both indoor and outdoor products such as televisions, computers, microwave ovens, photocopiers, lampshades, textiles, and furniture (Alaee et al., 2003; Segev et al., 2009). Due to the presence of a bromo substituent, these compounds are stable, bioaccumulate, and have a negative influence on the environment (Segev et al., 2009). Moreover, the identification of BFRs in various geographic areas, including the Arctic, is of concern (Segev et al., 2009). According to the literature, many chemical compounds belonging to BFRs exert adverse effects on ecosystems and living organisms (Van Cauwenbergh et al., 2020). The current continuous increase in the production of electrical products and, consequently, the increase in the amount of e-waste and the presence of BFRs in e-recycling samples poses a threat to both the environment and humans (Li et al., 2019; Luo et al., 2014). Moreover, classic BFRs such as polybrominated diphenyl ethers (PBDEs), tetrabromobisphenol A (TBBPA), and hexabromocyclododecane (HBCD) are characterized by bioaccumulation, ecotoxicity, hepatotoxicity, or neurotoxicity (Birnbaum & Staskal, 2004; Hendriks & Westerink, 2015; Segev et al., 2009; Szychowski & Wójtowicz, 2016). Therefore, substitutes of BFRs such as alternative flame retardants (AFRs) are being sought. Tris(2,3-dibromopropyl) isocyanurate (TBC or alternative abbreviation TDBP-TAZTO) is one of the group of novel flame retardants (NBFRs) comprising approximately 30 chemical compounds (Dong et al., 2021). NBFRs are characterized by similar physicochemical properties to traditional BFRs. However, due to the detection of large numbers of NBFRs in the environment, detailed analysis of the toxicity and ecotoxicity of such compounds is needed (Fig. 1).

TBC (molecular weight = 728.69 g/M) is a heterocyclic compound representing BFRs (basic physicochemical properties are shown in Table 1), which is used as an additive to materials in the production of electrical and electronic devices, musical instruments, and automotive components (Lörchner et al., 2019; Ruan et al., 2009). TBC produced as Armoquell FR930 by the Dutch company AkzoNobel Functional Chemicals is a white and/or off-white powder (Zhu et al., 2012). It is characterized by high durability, thermal stability, and resistance to photodegradation, which is related to the hexabrominated heterocyclic structure of s-triazine (Zhu et al., 2012). TBC has been used since 1959, when it was used in styrene polymers. Already in the 1990s, China estimated the annual production of TBC at over 500 metric tons, and an upward trend was forecast due to the increasing demand for electronic equipment and the withdrawal of some BFRs, e.g., penta- and octa-bromodiphenyl ether (BDE) (Dong et al., 2015; Li et al., 2011; Zhu et al., 2012).

**Table 1:** Tris(2,3-dibromopropyl) isocyanurate (TBC or TDBP-TAZTO) compared to classic BFRs (HBCD and PBDEs)—basic information according to Ruan et al. (2009)

Parameter	Value			
Name of the chemical compound	TBC	HBCD	PBDEs	
			Penta-BDE	Octa-BDE
CAS	52,434-90-9	3194-55-6	32,534-81-9	32,536-52-0
Water solubility (mg/L)	1.14 × 10^–5^	2.00 × 10^–5^	0.01	1.11 × 10^–8^
Vapor pressure (Pa, 25 °C)	1.57 × 10^–13^	2.23 × 10^–6^	1.44 × 10^–4^	1.69
Atmospheric oxidation half-life (days)	1.63	2.13	19.50	93.60
Octanol–water partition coefficient (log *K*_ow_)	7.37	7.74	7.66	10.33
Bioaccumulation factor (log BAF)/ Bioconcentration factor (BCF)	4.30	6211	3.69 × 10^4^	3
Octanol–air partition coefficient (log *K*_oa_)	23.68	11.80	11.15	15.85
Air–water partition coefficient (log *K*_aw_)	− 16.31	4.15	− 4.31	− 5.52

Given the increasing amount of data describing the detection of TBC in the environment and its effect on living organisms, the purpose of this paper is to summarize the current state of knowledge of the use, detection in the environment, and safety of TBC.

## Methods for TBC identification

High-performance liquid chromatography coupled with tandem mass spectrometry (HPLC—MS/MS) is the basic technique for detection of TBC in environmental samples, e.g., river water, soil, sediments, and in biological samples (earthworm and carp) (Ruan et al., 2009). The HPLC–MS/MS method was found to be effective for the simultaneous determination of TBC and HBCD with a TBC detection limit of 4.30 pg (Feng et al., 2010). Moreover, the evaluation of TBC in samples showed a concentration range of 136.10–5884.60 ng/g in the sediments and 51.10–1899.00 ng/g in fish *Cyprinus carpio* tissues (Feng et al., 2010). Huang et al. proposed the use of a bis-amino monomer to produce a TBC-MIP sensor (TBC imprinting poly-OPD-modified GCE) with a limit of detection of 62.60 pM (Huang et al., 2016). However, recent years have shown engagement of nanotechnology in the improvement in TBC detection methods. Feng et al. used a CdS-Mn/MoS_2_/CdTe/TiO_2_ quaternary photocatalyst based on a combination of 1D, 2D, and 3D nanostructures in an immunoassay to identify TBC in river water, with a linear range from 2.00 pM to 100.00 nM and a limit of detection 1.02 pM (Feng, Li, et al., 2018; Feng, Zhang, et al., 2018). In turn, Zhao et al. showed that the modification of the electrode with gold nanoparticles (AuNPs) and AgNO_3_, which was able to measure the electrochemiluminescence of TBC, caused a 20-fold reduction in the limit of detection, extending the linear range of the measurement up to five times (Zhao et al., 2011). Measurements of chemiluminescence and the application of nanotechnology were also reported by Tong et al., (2014) who used TiO_2_ nanotubes with CdTe/CdS quantum dots, achieving a limit of detection of 6.00 pM and a linear range from 10.00 pM to 50.00 nM, justifying the ability to detect the test compound (TBC) in water and soil samples (Tong et al., 2014). In a real-time immuno-PCR test with a DNA-gold-nanoparticles-based probe, the limit of TBC detection was 0.97 pg/L and the range of identification was from 0.10 pg/L to 0.10 ng/L (Bu & Zhuang, 2015). In turn, Shi et al. showed a linear range of 0.30–100.00 µg/L and a half maximal inhibitory concentration (IC_50_) value of 5.17 µg/L, with a recovery of 68–110% of TBC from biological samples: soil, water, and serum, using the competitive indirect enzyme-linked immunosorbent assay (ciELISA) with synthesized haptens (Shi et al., 2014). In a similar study, Feng et al. used a monoclonal antibody and performed the ciELISA test with TBC identification with the IC_50_ value = 1.59 µg/L, limit of detection (LOD) of 0.06 µg/L, and a precision range of 6.70–11.30% (Feng et al., 2014). In both studies, the ciELISA test values were consistent with UHPLC-MS/MS (Feng et al., 2014; Shi et al., 2014). Assessment of the presence of TBC in water from BFR-contaminated regions carried out with the use of ELISA with biotin and streptavidin amplification was found to be effective with a recovery of 92.10–109.20%. According to the results, the limit of detection and IC_50_ were 6.70 pg/mL and 0.66 ng/mL, respectively (Bu et al., 2014). The other methods used for TBC detection are based on an electrochemical immunosensor using competitive immunoreaction, with a detection limit of 8.60 nM and a range from 0.01 to 1 µM (Huang et al., 2014). Modifications of the methods were also related to the ways of identification of TBC in living organisms. In their study on the presence and distribution of the tested compound in mouse organs (liver, kidney, heart, and brain) administered at 160 mg/kg/day for 30 days, Tong et al., (2021) proved the effectiveness of matrix-assisted laser desorption/ionization imaging mass spectrometry (MALDI-IMS) on the matrix of 1,5-diaminonaphthalene hydrochloride and silver trifluoromethanesulfonate (NDA/AgOTf). The detection limit of the method was of the order of μg/mL (Tong et al., 2021).

The use of the above methods in tissue homogenates, in situ, and environment samples allows sensitive identification of TBC. However, due to the increasing demand and production of BFRs, it is necessary to constantly develop the scope of identification, LOD, modify the current methods, and develop methods for simultaneous determination of several BFRs in order to identify toxic compounds in the environment and biota quickly and precisely.

## Bioindicators of TBC

**Fig. 2:**
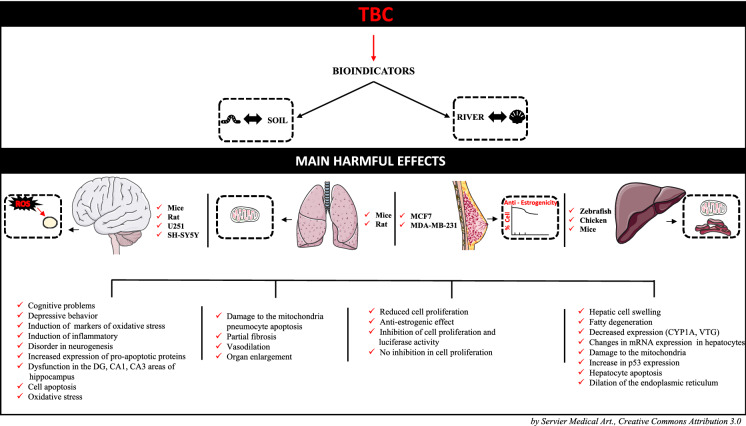
Identification and impact of TBC on living organisms

Mollusks are good indicators of water purity and have good resistance to persistent organic pollutants (POPs) and high bioaccumulation capacity due to low metabolic enzyme activity and high lipid content (Amoatey & Baawain, 2019) (Fig. 2). In addition, despite the differences in their anatomical structure, different species of mollusks constitute a promising bioindicator model due to their presence over a wide geographical range both in marine and in freshwater (Zhu et al., 2012). In a study conducted by Zhu et al. in China in 2009–2010, mollusks were selected to evaluate TBC content in organisms (Zhu et al., 2012). The frequency of TBC detection was 77%, and the concentration of TBC in the analyzed organisms ranged from below the detection limit (n.d.—not detected) up to 12.10 ng/g dry weight (dw). Interestingly, along with the higher trophic level of mollusks, the concentration of toxic chemical compounds decreased, which may indicate the opposite effect to biomagnification (i.e., the so-called trophic dilution) in the food chains of the aquatic ecosystem (Zhu et al., 2012) (Fig. 2). In addition, among the 11 species of Bohai sea mollusks used, *Mytilus edulis* turned out to be the most effective bioindicator due to the high accumulation capacity of the tested BFRs: TBC, HBCD, and PBDE (Zhu et al., 2012) (Fig. 2). As reported by Ruan et al., the soil earthworm is another TBC bioindicator (Fig. 2). The content of TBC in these organisms ranged from 9.75 to 78.80 ng/g dw (Ruan et al., 2009).

Bioaccumulation of chemical compounds in living organisms depends on many factors such as the rate of metabolic transformations, half-life in the tested organism, assimilation efficiency, seasonal differences in the concentration of chemical substances in the tested organisms, the presence of already suspended particles in the aquatic ecosystem, the growth dilution factor for individual species, and biotransformation of other BFRs, for example PBDE (Ruan et al., 2009; Zhu et al., 2012). Due to the number of variables influencing the accumulation of the tested compounds in living organisms, selection of an effective bioindicator for TBC requires further research and analysis of the mechanism and correlation of TBC absorption in individual organs depending on the concentration of the tested compound and variables in the surrounding environment.

## Detection of TBC in the environment

TBC was first identified in the environment in the vicinity of a factory in southern China in 2009 (Ruan et al., 2009). Ruan et al. showed the presence of the studied chemical compound in river water at a concentration of 2.33–163.00 ng/L in surface sediments in the range from 85.00 ng/g to 6.03 µg/g dry weight (dw) and in soil in the range from 19.60 to 672.00 ng/g dw (Ruan et al., 2009). The physicochemical properties of TBC and its ability to spread are similar to those of penta-BDE and HBCD. The high values of the octanol–water partition coefficient (Kow) and the octanol–air partition coefficient (Koa) indicated the possibility of TBC migration over long distances. Moreover, with an increasing distance from the source of TBC emission, the content of the compound in the soil decreased (Ruan et al., 2009). As shown by Wang et al. in a study carried out in the southeastern part of Beijing (China), the incidence of TBC detection in agricultural soils increased significantly and amounted to 25% in 2010 and 100% in 2011, which may be related to urbanization and increasing production (Wang et al., 2013). The median level of the compound tested was 0.19 ng/g dw, and its range was from below the detection limit up to 1.62 ng/g dw. In addition, there was no effect of the various sources of soil irrigation used on the TBC content (Wang et al., 2013). In the industrialized area of Ningbo City in eastern China, Tang et al. demonstrated the presence of TBC in surface soils at concentrations ranging from below the detection limit to up to 16.40 ng/g dw, with a median of 0.95 ng/g dw and detection frequency of 57.80%. The highest concentrations of TBC came from waste dumping sites (8.10 ± 1.71 ng/g dw, n = 6) and industrial areas (4.51 ± 0.99 ng/g dw, n = 22), compared to lower concentrations in traffic areas, vegetable soils, residential areas, and farmland soils (Tang et al., 2014). Wetlands can act as natural filters for the aquatic ecosystem; hence, it is important to study the sediments in these areas, as in the case of the wetlands of the Yellow River Delta in Dongying (China) (Wang et al., 2017). The presence of TBC was detected in the concentration range from 0.20 to 29.03 ng/g dw, with a mean value of 6.88 ng/g dw. The geographic distribution of the compound was high in the vicinity of the polluted river and the tourist center area and lower along the old course of the Yellow River, which is protected by the government (Wang et al., 2017). The analysis of the TBC pollutant in a collection of sediments from 25 sites in Jiaozhou Bay wetland in China revealed the concentration of the tested compound in the range from 1.20 to 8.76 ng/g dw and the mean concentration and median of 3.60 ng/g dw and 2.50 ng/g dw, respectively (Wang et al., 2016). The overall level of TBC from the Xiaojianxi refuse landfill to the bay tidal zone was found to decrease (Wang et al., 2016). The presence of TBC was also detected by Zhang et al. in sediments from the intertidal zone of the New River estuary in Beidaihe Wetlands, China, at concentrations ranging from 2.78 to 35.10 ng/g dw, with a mean value of 13.40 ng/g dw. Higher TBC values were identified in the lower part of the intertidal zone tides than in the upper part (with abundant vegetation), which may have been caused by the oil spill (Zhang et al., 2018). TBC was also identified by Feng et al. in seawater and sediments in the South China Sea. The range and frequency of detection were n.d. up to 2.12 ng/L and 36.84%, respectively, in the seawater and n.d. up to 0.22 ng/g dw and 88.89%, respectively, in the sediments (Feng et al., 2021). The summary of the identification of TBC in the environment is presented in Table 2.

**Table 2:** Detection of TBC in environmental samples. n.d.—not detected; dw—dry weight

Area	Environmental sample type	Concentration range	Median/mean concentration	References
Liuyang River	River water	2.33 to 163.00 ng/L	–	(Ruan et al., 2009)
Liuyang	Sediment	85.00 ng/g to 6.03 µg/g dw	–	(Ruan et al., 2009)
Liuyang Hunan Province (China)	Soil	19.60 to 672.00 ng/g dw	–	(Ruan et al., 2009)
Tongzhou region near Liangshui River (Beijing, China)	Soil	n.d. up to 1.62 ng/g dw	0.19 ng/g dw/-	(Wang et al., 2013)
Ningbo region, Zhejiang Province, (China)	Soil	n.d. up to 16.40 ng/g dw	0.95 ng/g dw/-	(Tang et al., 2014)
Yellow River Delta	Sediments	0.20 to 29.03 ng/g dw	− 6.88 ng/g dw	(Wang et al., 2017)
Jiaozhou Bay wetland in Qingdao, Shandong Province (China)	Sediments	1.20 to 8.76 ng/g dw	2.50 ng/g dw/3.60 ng/g dw	(Wang et al., 2016)
Intertidal zone New River Estuary Beidaihe Wetlands (Hebei, China)	Sediments	2.78 to 35.10 ng/g dw	− 13.40 ng/g dw	(Zhang et al., 2018)
South China Sea	Seawater	n.d. up to 2.12 ng/L	–	(Feng et al., 2021)
South China Sea	Sediments	n.d. up to 0.22 ng/g dw	< 0.059 ng/g dw	(Feng et al., 2021)

Due to the increasing amount of e-waste, a study in China found the highest average concentration of TBC in dust from e-recycling plants (2060 ng/g) (Shen et al., 2019). TBC was also present inside nearby residential buildings (33.20 ng/g) and on the street surface (18.80 ng/g) (Shen et al., 2019). Moreover, the tested TBC was considered the main pollutant in e-waste, in addition to PBDE, TBBPA and decabromodiphenyl ethane (DBDPE), which proves the considerable role of the compound (Shen et al., 2019). In a study conducted in North America, Guo et al. showed the absence of TBC in e-waste air, e-waste dust, house dust, and environmental samples (Guo et al., 2018). In addition, Miyake et al. detected the presence of TBC in fireproof curtains purchased in Japan (Miyake et al., 2017). As shown by Tourinho et al., plastic is the main material among marine litter consumed by sea turtles and birds (Tourinho et al., 2010). The threat posed by TBC to the marine ecosystem was analyzed by assessing the adsorption of this compound on microplastic in stimulated seawater, showing the most effective adsorption in equilibrium conditions of 15 h, temperature 15 °C, and 14% salinity (Liu et al., 2018). Particle size is equally important, as the adsorption effect increases with a decreasing particle size (Liu et al., 2018).

The described flame retardant was first identified in environmental (soil, water, sediment) and biological (carp, earthworm) samples in China in 2009 (Ruan et al., 2009). It is still ongoing, which is confirmed by the detection of TBC in environmental samples (water, sediment) also in China in 2021 (Feng et al., 2021). The concentration of TBC research in China, which is a major producer and consumer of BFRs, may be related to the continuous development of urbanization and economy of the area (Tang et al., 2014); moreover, China is a major generator of e-waste, which is a cluster of compounds of various FRs (Li et al., 2019; Luo et al., 2014; Zheng et al., 2015). In addition to the physicochemical parameters of TBCs such as the high log *K*_ow_ value resulting in easy absorption into sediment (Wang et al., 2016), the influence of such environmental factors as pH and temperature on the properties and accumulation of BFRs in the environment is also important (Segev et al., 2009). Therefore, a comparative analysis of TBC accumulation in other geographic areas would be valuable, especially in Europe and both North and South America.

## Impact of TBC on living organisms

Various living organisms are characterized by different transport, metabolism, and biotransformation of TBC (Zhang et al., 2011). Moreover, due to the differences in the chemical structure and physicochemical properties, compounds belonging to the BFR group are characterized by a different absorption route and metabolism of chemical compounds (De Wit, 2002). Both the molecular weight and the log *K*_ow_ of the substance have a significant influence on the assimilation of compounds into the bloodstream of animals and/or humans (Gülden et al., 2002; Hendriks & Westerink, 2015). A good and sensitive marker of the presence of xenobiotics in the tested organisms is the CYP1A family of cytochrome P450 (Zhang et al., 2011). TBC has a high value of the bioaccumulation factor (BCF) (Ruan et al., 2009); therefore, it is important to assess the effect of this chemical on living organisms (Table 3).

**Table 3:** Summary of literature data on the influence of TBC on living organisms

Model	Tested material/parameter	TBC impact/results	References
Microalgae *Nannochloropsis* sp.	Growth	Inhibition growth	Wang et al., (2014)
	Photosynthetic genes (*psa*A, *psb*C)	Decreased expression of *psa*A, *psb*C	
Zebrafish (*Danio rerio*)	Growth	No changes	Zhang et al., (2011)
	Survival	No changes	
	Liver	Hepatic cell swelling	
		Fatty degeneration	
		Decreased expression of *CYP1A* and *VTG*	
	Gills	Proliferation of epithelial cells	
		Swelling of epithelial cells	
	Intestines	Proliferation of goblet cells	
	Testicles	Cell-free areas	
	Ovaries	Thinly scattered vitellogenic granules	
Zebrafish (*Danio rerio*) larvae	Early organogenesis	No changes	Li et al., (2011)
	Gas bladder	Impaired filling: changes in cytoplasmic vesicles, mitochondrial	
Chicken (*Gallus gallus domesticus*)	Liver (embryonic hepatocytes)	Thyroid endocrine pathway (decrease in *IGF1* expression)	Porter et al., (2014)
		Sex steroid pathway (increased *VTG2*)	
		Redox balance (*TXN* regulation)	
Mice BALB/c strain	Brain (hippocampus)	Depressive behavior	Dong et al., (2015)
		Induction of markers of oxidative stress	
		Increased expression of pro-apoptotic proteins	
	Liver	Damage to mitochondria	Li et al., (2015)
		Increase in p53 expression	
		Hepatocyte apoptosis	
		Dilation of the endoplasmic reticulum	
	Lung	Damage to mitochondria Pneumocyte apoptosis	
	Kidneys	No significant changes	
	Spleen	No significant changes	
Rat	Primary cultures of CGN	Cytotoxic effect on developing CGN (decreased cell viability)	Qu et al., (2011)
Rat Sprague**–**Dawley strain	Brain	Increase in the level of MDA, NO, and H_2_O_2_	Feng, Li, et al., (2018); Feng, Zhang, et al., (2018)
		Cognitive problems	Ye et al., (2015)
		Depressive behavior	
		Induction of markers of oxidative stress	
		Induction of inflammatory conditions	
		Disorder in neurogenesis	
		Increased expression of pro-apoptotic proteins	
		Dysfunction in the DG, CA1, and CA3 areas of the hippocampus	
Rat Wistar strain	Activity	No changes	Zhou et al., (2018)
	Growth	No changes	
	Body weight	No changes	
	Food consumption	No changes	
	Lung	Diffuse, thickened epithelial walls	
		Partial fibrosis	
		Vasodilation	
	Spleen	Organ enlargement	
	Kidney	Minor congestion	
		Inflammation	
	Serum	Decrease in the level of free T4	
		Decrease in the level of estradiol	
U251 cell line	Neurotoxicity	Cell apoptosis	Feng, Li, et al., (2018); Feng, Zhang, et al., (2018)
		Increase in the level of MDA-(lipid peroxidation)	
SH-SY5Y cell line	Neurotoxicity	Cell apoptosis	Feng, Li, et al., (2018); Feng, Zhang, et al., (2018)
		No changes in the level of MDA	
		Cell apoptosis	Dong et al., (2015)
		Oxidative stress	
	Neuronal differentiation	Increase in the ROS level	Szychowski et al., (2021)
		Increased caspase activity 3	
		Greater sensitivity to TBC of differentiated cells	
		Disruption of AhR and *CYP1A1* mRNA expression in differentiated cells	
		Decrease in EROD activity with differentiation of cells into neurons	
		Lack of *CYP2B6* mRNA expression	
MCF-7 cell line	Endocrine function	Reduced cell proliferation	Krivoshiev et al., (2016)
		Anti-estrogenic effect	
		Inhibition of E_2_-induced cell proliferation	Cao et al., (2018)
		Inhibition of E_2_-induced luciferase activity	
H295R cell line	Endocrine function	Inhibition of E_2_-induced luciferase activity	Li et al., (2016)
		Decrease in E_2_ and testosterone levels	
		No change in the expression of steroid genes	
		No cytotoxic effect	
		Inhibition of the biosynthesis of steroid hormones	
MDA-MB-231 cell line	Endocrine function	No inhibition in cell proliferation	Cao et al., (2018)
Humans	Skin absorption	Higher TBC absorption	Shen et al., (2019)
	Absorption by swallowing	Lower TBC absorption	

### Water organisms

Algae are water purity indicators; moreover, they are used as food for aquatic organisms, e.g., larvae, young mollusks, and larvae of selected crustaceans and fish, influencing their growth and development (Brown & Jeffrey, 1992; Wang et al., 2014). The presence of biotic stressors influences the photosynthesis process in plants, for example by reducing the expression of genes involved in photosynthesis. By regulating photosynthetic proteins, the plant engages in a defense mechanism (Bilgin et al., 2010). Therefore, it is necessary to assess the impact of the transferred TBC on aquatic ecosystems. Wang et al. investigated the effect of TBC at concentrations of 10, 100, and 1000 ng/mL on a selected family of microalgae, i.e., *Nannochloropsis* sp., and demonstrated inhibition of their growth. Additionally, the authors observed a decrease in the expression of the *psa*A and *psb*C genes encoding proteins crucial for the photosystem along with the increase in the concentration used (Wang et al., 2014).

The zebrafish (*Danio rerio*) has been found to be a suitable model organism to analyze the mechanism of action and toxicity of BFR (Usenko et al., 2016). As shown in the research, TBC disrupts the proper functioning of *Danio rerio* (Li et al., 2011; Zhang et al., 2011)*.* To test the effects of TBC on fish growth, survival, tissue function, hormone levels, enzyme activity, and gene expression, Zgang et al. studied *Danio rerio* for 28 days of exposure to TBC at concentrations of 0.25, 1, and 4 mg/L (Zhang et al., 2011). No changes in the growth and survival of the tested organism were observed (Zhang et al., 2011). However, TBC caused changes in the liver, gills, and intestines, and the observed pathologies were similar in both males and females (Zhang et al., 2011). In the liver, hepatic cell swelling and fatty degeneration were detected as well as decreased expression of the cytochrome *P4501a* (*Cyp1a*) and vitellogenin (*Vtg*) genes (Zhang et al., 2011). In addition, TBC exerted an effect on the proliferation and swelling of epithelial cells in the gills, proliferation of intestinal goblet cells at a concentration of 4 mg/L, cell-free areas in the testes, and changes in vitellogenic oocytes (Zhang et al., 2011). Summarizing, the described results emphasize the undesirable effect of TBC on the reproductive and endocrine systems in *Danio rerio* (Zhang et al., 2011). In contrast, evaluation of gas bladder filling in zebrafish larvae exposed to TBC showed impairment that had the strongest effect on the larvae 72–96 h after fertilization and coincided with the time of the first gas bladder filling (Li et al., 2011). Therefore, the observed changes in the density of cytoplasmic vesicles and the mitochondrial defect through the secretion of a mucus-like substance cause impaired filling of the organ and, as a result, may lead to disturbances in the motility of fish larvae and death due to starvation (Li et al., 2011). The carp (*Cyprinus carpio*) was another organism inhabiting aquatic ecosystems in which TBC contamination was studied (Ruan et al., 2009). Evaluation of the TBC levels showed a tendency to accumulate in brain and adipose tissue, which are fat-rich organs in *Cyprinus carpio*. Moreover, the tendency to accumulate in the brain may indicate the potential for TBC to pass through the blood–brain barrier (Ruan et al., 2009). Lower values of the analyzed compound were observed in muscles and gills, while the content in the liver covered a wide range, which was supposed to be the effect of TBC metabolism. The important role of the food chain as a significant source of TBC in fish should also be emphasized, which is associated with the low concentration of the compound in the gills and its high levels in the intestines (Ruan et al., 2009).

### Terrestrial organisms and in vitro studies

The effect of TBC was also studied in terrestrial ecosystems, including birds. A study conducted by Porter et al. demonstrated the greatest effect of TBC and tris(1,3-dichloro-2-propyl) phosphate (TDCPP), among 16 FRs tested, on the mRNA expression of selected genes in avian embryo hepatocytes (Porter et al., 2014). The exposure to TBC caused a change in the gene expression of the thyroid hormone pathway, including a 28.60-fold decrease for the insulin-like growth factor 1 gene (*IGF1*) (Porter et al., 2014). Moreover, changes were also observed in the sex steroid pathway, i.e., increased vitellogenin 2 (*VTG2*) expression and upregulation of thioredoxin (*TXN*), which is responsible for the oxidative balance (Porter et al., 2014).

As shown in other research, exposure to TBC had an adverse effect on the organism of rodents (Dong et al., 2015; Feng, Li, et al., 2018; Feng, Zhang, et al., 2018; Li et al., 2015; Qu et al., 2011; Ye et al., 2015). In rats exposed to TBC at concentrations of 0.50 mg/kg or 2 mg/kg body weight, analysis of oxidative stress in the brain showed greater toxicity of TBC at a concentration of 0.50 mg/kg and an increased level of malondialdehyde (MDA), nitric oxide (NO), and hydrogen peroxide (H_2_O_2_) (Feng, Li, et al., 2018; Feng, Zhang, et al., 2018). The levels of total superoxide dismutase (T-SOD) and reduced glutathione (GSH) almost did not change, which may be caused by the insufficient exposure time (7 days). The authors documented well that TBC induced oxidative stress, and changes caused by TBC were predicted to be reversible by the use of antioxidants (Feng, Li, et al., 2018; Feng, Zhang, et al., 2018). In a study carried out on rats exposed to 5 and 50 mg/kg TBC administered by gavage for 6 months, Ye et al. assessed the effect of the tested compound on the nervous system. Numerous disorders were shown, e.g., cognitive problems and depressive behavior, which were most likely the result of hyperactivation of the hypothalamic–pituitary–adrenal axis by the tested chemical, induction of oxidative stress evidenced by the increasing levels of markers of oxidative stress, and a decrease in antioxidant enzymes (Ye et al., 2015). Other symptoms of rat TBC treatment in the nervous system were inflammation, disturbances in neurogenesis and neuroplasticity, induction of apoptosis directly or through the activation of astrocytes, and pathologies in the DG, CA1, and CA3 areas of the hippocampus (Ye et al., 2015). As reported by Dong et al., a monthly treatment of mice with 5 or 50 mg/kg TBC induced oxidative stress in mouse hippocampal neurons, decreased the levels of Bcl-2 protein, and increased the level of Bax protein, i.e., a marker of activation of apoptosis (Dong et al., 2015). In addition, TBC at a concentration of 5–10 µM showed a cytotoxic effect on cultures of developing rat cerebellar granular neurons (CGNs), whereas no changes were detected in mature CGNs (Qu et al., 2011). There were also differences in the liver, respiratory system, and hormone levels, in addition to the above-described changes in the nervous system of rodents (Li et al., 2015; Zhou et al., 2018). In the liver, the expression of the *p53* gene, which plays a role in the convergence of external and internal apoptotic pathways, was increased (Haupt et al., 2003; Li et al., 2015). Furthermore, mitochondrial abnormalities were most likely responsible for the damage to the mouse liver and lung induced by TBC (Li et al., 2015). The major changes shown by Zhou et al. were lung damage and significant reductions in the thyroid hormone thyroxine (T4) and estradiol levels as a result of 4-week exposure of rats to TBC at 2 mg/kg/day TBC and 50 mg/kg/day TBC, respectively (Zhou et al., 2018).

The effect of TBC on the generation of oxidative stress was tested on the human malignant glioma U251 cell line and the neuroblastoma SH-SY5Y cell line. The analysis showed greater neurotoxicity of TBC to the U251 cell line through lower cell viability and increased apoptosis and oxidative stress markers than to the SH-SY5Y cells (Feng, Li, et al., 2018; Feng, Zhang, et al., 2018). However, Dong et al. showed the neurotoxic effect of TBC on the SH-SY5Y cell line in 48-h exposure to TBC at concentrations of 12.50, 25, and 50 µM. Dong et al. hypothesized that the generated reactive oxygen species (ROS) caused increased expression of pro-apoptotic proteins (Dong et al., 2015). In turn, Szychowski et al. showed that the sensitivity of SH-SY5Y cells to TBC depended on the degree of their differentiation. Treatment of cells with TBC for 24 h induced an increase in ROS levels in undifferentiated and 7- and 14-day-old differentiated SH-SY5Y cells; the highest increase was found for 7-day-old differentiated cells. In turn, an increase in caspase-3 activity was shown in the 14-day-old differentiated cells at all TBC concentrations in the range of 1 nM-100 µM (Szychowski et al., 2021). Moreover, to analyze the activity of the CYP1A1 enzyme, Szychowski et al. performed a fluorescence test using ethoxyresorufin-O-deethylase (EROD). The authors also described that the aryl hydrocarbon receptor (*AhR*) and the *CYP1A1* mRNA expression in the differentiated SH-SY5Y cells were disrupted by TBC. The EROD activity decreased after the TBC treatment of the SH-SY5Y cells during differentiation into neurons (Szychowski et al., 2021). Additionally, the lack of *CYP2B6* mRNA expression should be emphasized, which suggests the absence of CAR receptor involvement in TBC action (Szychowski et al., 2021).

The role of TBC in changes in the functioning of the endocrine system in the MCF-7 cell line of estrogen-dependent breast adenocarcinoma treated with 17β-estradiol (E_2_) was investigated as well, and an anti-estrogenic effect of TBC was indicated (Krivoshiev et al., 2016). TBC reduced the proliferation of the MCF-7 cells by 22.90% with IC_20_ (concentration at 20% inhibition) and relative inhibitive potency (RIP) values of 62.20 µM and 1 × 10^–5^, respectively (Krivoshiev et al., 2016). The synthesis of steroid hormones takes place in gonads and suprarenal glands (Li et al., 2016). Forskolin is a diterpenoid capable of modulating the adenylate cyclase enzyme and cyclic adenosine monophosphate (cAMP) levels; moreover, it is also applied as a modulator of glucocorticoid and aldosterone synthesis (Purdy et al., 1991). In addition, it was shown that the treatment of the H295R adrenocortical cancer cell line with forskolin resulted in a more similar gene expression profile to that in normal human adrenal gland cells than without the use of forskolin (Oskarsson et al., 2006). The evaluation of the effect of TBC in concentrations of 1 nM-1 µM on the H295R cell line in both the presence and absence of exposure to 10 µM forskolin for 72 h revealed no cytotoxic effect. The differences found were the reduction in the level of steroid hormones such as E_2_ and testosterone through the exposure to TBC as well as TBC and forskolin. Despite the lack of changes in the expression of the *StAR, 3bHSD2, CYP17, CYP21, CYP19, HMGR, 17bHSD1, 17bHSD4,* and *CYP11A* steroid genes under the influence of TBC, the use of forskolin increased the expression of the tested genes except for *HMGR, 17SD1,* and *17bHSD4*. In addition, the exposure of the H295R cell line to TBC induced by forskolin led to inhibition of *StAR, 3bHSD2, CYP17, CYP21*, and *CYP19* mRNA expression and, consequently, to inhibition of steroid hormone biosynthesis (Li et al., 2016). The anti-estrogenic effect of TBC was also demonstrated by inhibition of E_2_-induced proliferation of estrogen-dependent MCF7/BUS human breast cancer cells (1 nM to 10 µM TBC) and luciferase. It should be noted that the anti-estrogenic mechanism of TBC may involve the estrogen receptor alpha (ERα) pathway, as confirmed by the lack of an effect of TBC and E_2_ on the inhibition of proliferation of the ERα-negative human breast cancer cell line MDA-MB-231 (Cao et al., 2018). As shown by the analyses, TBC differs in its structure from the traditional agonists and antagonists of ERα, e.g., E_2_ and 4-hydroxytamoxifen (OHT), which have an OH group involved in binding the ligand to ERα. Molecular modeling showed that, due to the high molecular volume and intermolecular collisions, the probability of TBC binding in the ERα pocket bound by E_2_ or OHT is low. Ultimately, based on molecular docking and molecular dynamics, it was found that TBC has an affinity for the AF-2 ERα LBD (ligand-binding domain) complex. Furthermore, E2-Erα-TBC was reported to interfere with the recruitment of cofactors in the estrogen pathway (Cao et al., 2018).

As reported by Shen et al., employees of e-waste plants were the most exposed to TBC in dust from e-waste, followed by children, while the lowest exposure was shown for adults. Larger amounts of TBC dust were absorbed into the body through skin absorption than ingestion (Shen et al., 2019). Examples of bioindicators and major damage from exposure to TBC are summarized in Fig. 2.

According to the above studies, TBC has a negative effect on biota. Therefore, it is necessary to control the accumulation and spread of this compound in food chains regularly. The current methods of analysis have elucidated the presumed mechanism of action of TBC in the nervous system, but it is necessary to understand the mode of action and biotransformation of the compound in other tissues.

## TBC degradation methods

TBC has been placed on the Environment Canada screening list as a chemical with a lower ecological hazard and has been recommended for further research by the UK Environment Agency (Tang et al., 2014; Wang et al., 2013). BFRs, including TBCs, are persistent, accumulate in the environment, and have limited biodegradability (Segev et al., 2009). The fate of test chemicals and environmental transport is influenced by many abiotic and biotic environmental processes that can either reduce or increase the toxicity of BFRs. The group of abiotic processes includes photodegradation, wet and dry deposition, high-temperature decomposition, and reactions with various other compounds and/or radicals present in the environment, which affects TBC degradation (Segev et al., 2009). In turn, the group of biotic processes includes bioaccumulation, biotransformation, and biodegradation (Segev et al., 2009). Due to the potential danger of TBC to the environment and living matter, studies were carried out on methods for degradation of the compound (Liang et al., 2016; Lörchner et al., 2019). In analyses carried out in an aqueous system, Liang et al. demonstrated more efficient photolysis of TBC by UV radiation than Xe lamp-generated radiation; the former method caused 95% decomposition of TBC contaminants at a concentration of 10 μM after 120 min of UV irradiation (Liang et al., 2016). In addition to the higher efficiency of TBC degradation by UV-C irradiation demonstrated by Lörchner et al., compared to the lower effectiveness of simulated sunlight, the half-life and photolytic degradation rate relative to first-order kinetics were *t*_1/2_ = (17 ± 2) min and *k* = (41 ± 5 × 10^–3^) min^−1^, respectively. Moreover, as shown by the results, de-bromination, hydroxylation, and dehydrogenation were the main pathways of TBC degradation (Lörchner et al., 2019). Further analysis of the fate of TBCs is required taking into account biochemical transformation, degradation, and the role of microorganisms and/or derivatives responsible for the production of intermediates (Segev et al., 2009).

## Conclusions

This review is the first summary of the current state of knowledge of the presence of TBC in environmental samples and living organisms as well as the mechanism of its action in cells. Currently, TBC, which was supposed to be an alternative to classic BFRs, turns out to be an equally serious problem for the environment and living organisms. TBC is widely detected in soil, sediments, river water, and such materials as microplastic, curtains, and e-waste devices. This compound has strong potential to accumulate in organisms and has been detected in tissues of a number of species; for instance, it can easily penetrate the brain. According to the presented literature, TBC causes damage mainly to the nervous and endocrine systems, lungs, and liver and impairs the function and development of the reproductive system. To date, scientists agree that TBC acts with involvement of ERα and AhR receptors. Moreover, due to the known cross talk between these receptors, their interaction cannot be excluded in the TBC mechanism of action. Most importantly, since TBC interferes with the correct expression and/or activity of P450 enzymes, it may interfere with the proper metabolism of other xenobiotics. Given the many disadvantages of TBC and its accumulation in living organisms affecting their function, we propose that new and safe alternatives to BFRs should be sought.

## Abbreviations

AFRs: Alternative flame retardants
AuNPs: Gold nanoparticles
BCF: Bioconcentration factor
BFRs: Bromine flame retardants
CGN: Cerebellar granular neurons
ciELISA: Competitive indirect enzyme-linked immunosorbent assay
*CYP1A*: Cytochrome *P4501A*;
DBDPE: Decabromodiphenyl ethane
dw: Dry weight
E_2_: Estradiol
FR: Flame retardants
GSH: Reduced glutathione
HBCD: Hexabromocyclododecane
HPLC–MS/MS: Liquid chromatography coupled with tandem mass spectrometry
H_2_O_2_: Hydrogen peroxide
H295R: Adrenocortical cancer cell line
IC_20_: Concentration at 20% inhibition
IC_50_: Half maximal inhibitory concentration
*IGF1*: Insulin-like growth factor 1 gene
log *K*_aw_: Air–water partition coefficient
Koa: Octanol–air partition coefficient
Kow: Octanol–water partition coefficient
LBD: Ligand-binding domain
LDH: Lactate dehydrogenase
LOD: Limit of detection
MALDI-IMS: Matrix-assisted laser desorption/ionization imaging mass spectrometry
MCF-7: Cell line of estrogen-dependent breast adenocarcinoma
MDA: Malondialdehyde
MDA-MB-231: Breast cancer cell line
NBFRs: Novel flame retardants
n.d.: Not detected
NDA/AgOTf: Matrix of 1,5-diaminonaphthalene hydrochloride and silver trifluoromethanesulfonate
NO: Nitric oxide
OHT: 4-Hydroxytamoxifen
PBDE: Polybrominated diphenyl ethers
POP: Persistent organic pollutant
RIP: Relative proliferative potency
ROS: Reactive oxygen species
SH-SY5Y: Neuroblastoma cell line
TBBPA: Tetrabromobisphenol A
TDBP-TAZTO/TBC: Tris(2,3-dibromopropyl) isocyanurate
TDCPP: Tris(1,3-dichloro-2-propyl) phosphate
T-SOD: Total superoxide dismutase
TXN: Thioredoxin
T4: Thyroxin
U251: Malignant glioma cell line
VTG: Vitellogenin
VTG2: Vitellogenin 2
